# Beyond mental well-being: A One Health perspective on biophobias

**DOI:** 10.1093/biosci/biaf084

**Published:** 2025-06-25

**Authors:** Stefano Mammola, Veronica Nanni, Simone Martino, Ricardo Correia, Ester M Eckert, Melissa M Norberg, Masashi Soga

**Affiliations:** Molecular Ecology Group, Water Research Institute, National Research Council in Verbania, Italy; Finnish Museum of Natural History, University of Helsinki, Helsinki, Finland; National Biodiversity Future Center, Palermo, Italy; Molecular Ecology Group, Water Research Institute, National Research Council, Verbania, Italy; Science, Technology, and Society Department, School for Advanced Studies, Pavia, Italy; James Hutton Institute, Aberdeen, Scotland; Biodiversity Unit of the University of Turku, Turku, Finland; Helsinki Lab of Interdisciplinary Conservation Science, Department of Geosciences and Geography, University of Helsinki, Helsinki, Finland; Helsinki Institute for Sustainability Science, University of Helsinki, Helsinki, Finland; Molecular Ecology Group, Water Research Institute, National Research Council, Verbania, Italy; National Biodiversity Future Center, Palermo, Italy; Lifespan Health and Wellbeing Research Centre, Macquarie University, Macquarie Park, New South Wales, Australia; Graduate School of Agricultural and Life Sciences, University of Tokyo, Tokyo, Japan

**Keywords:** biophilia, ecoanxiety, ecophobia, human–wildlife conflict, specific phobia

## Abstract

Aversive responses toward elements of the natural world, termed *biophobias*, are widespread. Whether they involve an irrational fear of animals, plants, germs, or dark forests, biophobias have far-reaching consequences that remain largely unstudied outside psychology and psychiatry. Foremost, biophobias affect mental health and entail direct (e.g., healthcare) and indirect (e.g., absenteeism from work) costs. In addition, they contribute to environmental and health issues through the overuse of pesticides and sanitizers, hinder sustainability efforts (e.g., insect phobia as a barrier to adopting insects in Western diets), and incur nonmaterial costs such as cultural erosion and avoidance of nature. Because these impacts emerge from complex interactions between human societies and ecosystems, we argue that biophobias are a quintessential One Health issue. One Health thinking could guide research and policy efforts to integrate medical, socioeconomic, and ecological perspectives in addressing biophobias. To advance a One Health agenda for biophobias, key knowledge gaps urgently need to be addressed.

If you have ever been unnerved by a spider, steered clear of dense dark forests, or refrained from swimming in crystal-clear waters for fear of sharks, you are not alone. Aversive responses toward elements of the natural world, known as *biophobias* (figure 1), are widespread (Soga et al. 2023, Soga and Evans 2024). Biophobias can be broadly defined as feelings of fear of, anxiety about, or disgust toward specific living organisms, biotic elements of nature, or related stimuli that pose little to no harm but elicit avoidance, often with detrimental effects. In some cases, biophobias may meet criteria for a psychological disorder (Norberg et al. 2024). Some of the most common biophobias involve animals (Correia and Mammola 2024, Zeller et al. 2025), with as many as 22% of individuals irrationally fearing insects, spiders, and other taxa (Eaton et al. 2018). Beyond animals, biophobias encompass the fear and avoidance of other living elements, such as germs, plants, mushrooms, and dense forests (Correia and Mammola 2024). Although the exact prevalence of different biophobias remains unclear, global urbanization and reduced direct exposure to nature are thought to contribute to their increase (Fukano and Soga 2021, Soga et al. 2023, Correia and Mammola 2024).

**Figure 1.**
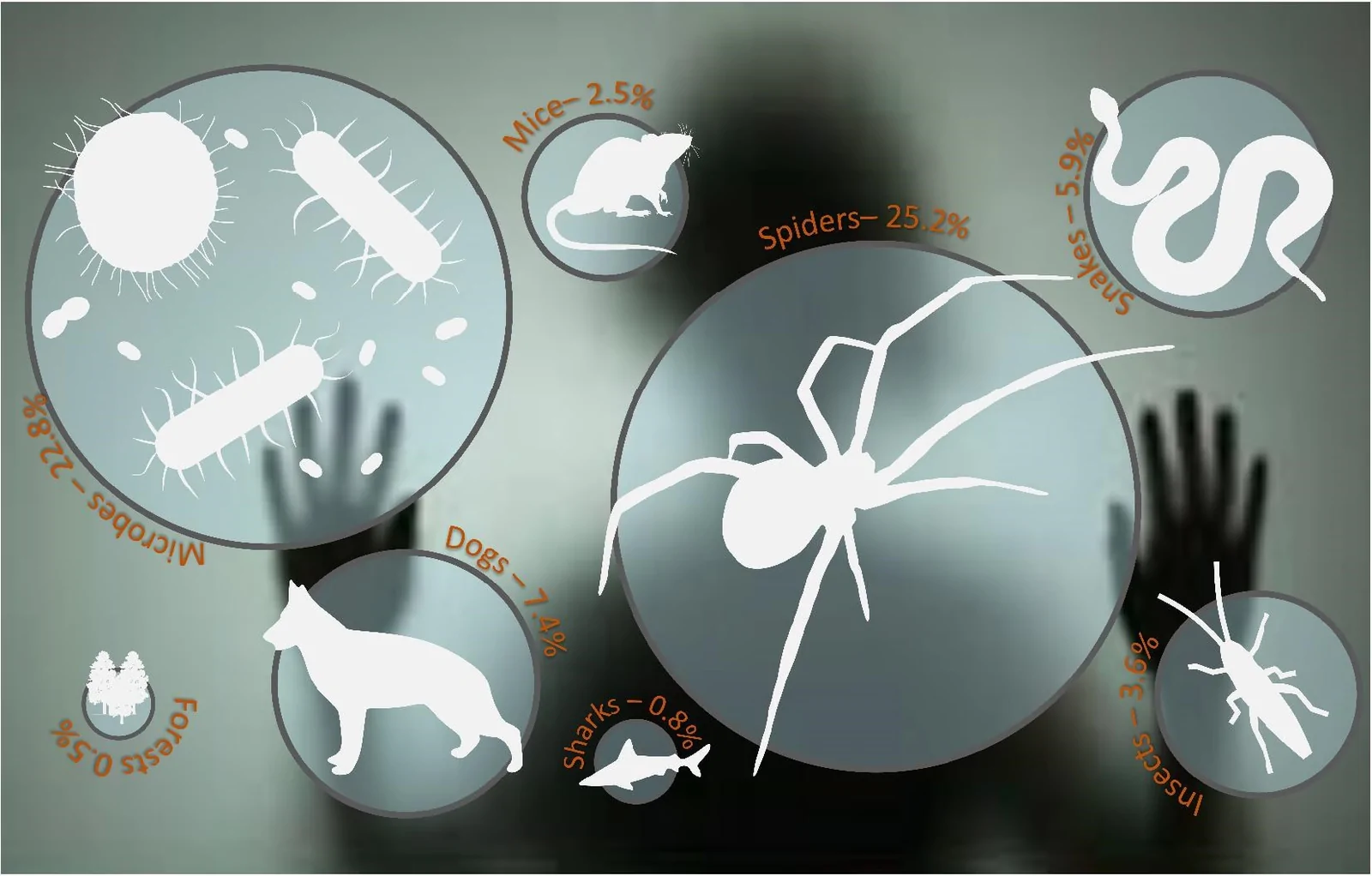
The relative prevalence of different biophobias. Relative prevalence of phobia of spider (arachnophobia), germs (mysophobia), dogs (cynophobia), snakes (ophidophobia), insects (enthomophobia), mice (musophobia), sharks (selacophobia) and forests (hylophobia) as indirectly estimated through internet searches. Percentages represent the relative proportion of internet searches conducted through Google's search engine for each type of biophobia considered, as was reported by Correia and Mammola (2024). The search volume for each biophobia was obtained using “topic” searches in Google Trends, which aggregate multiple related terms, including synonyms (e.g., “arachnophobia” or “fear of spiders”) and translations in different languages (e.g., “fear of spiders” or “*medo de aranhas*”). Note that the percentages do not sum to 100%, because only a subset of the biophobias analyzed by Correia and Mammola (2024) is shown for illustrative purposes. Images: *Latrodectus hasselti* by Cathy (CC BY-SA 3.0); all other silhouettes and figures used are under public domain (CC0).

Although a certain level of fear may be helpful in some situations, widespread avoidance, as is the case with biophobia, can have tangible implications that extend beyond personal discomfort (Soga and Evans 2024). Biophobias affect mental health and, when they are severe, can affect those around the sufferer as well (Norberg et al. 2024). Unfortunately, individuals with a biophobia, particularly children, are less likely to seek treatment than those with non-nature-related specific phobias (Essau et al. 2000, Wardenaar et al. 2017). People are unlikely to seek out treatment when they do not recognize an irrational fear as senseless, even when it is accompanied by significant avoidance or impairment in personal, social, or occupational or academic functioning, warranting a specific phobia diagnosis (Grenier et al. 2011). Plus, it can be difficult to obtain effective treatment for a phobia, because many therapists incorrectly believe the gold standard treatment—exposure therapy (see box 1)—to be risky and lack sufficient training to deliver it (Trivasse et al. 2020).

Box 1. **Medical treatment of biophobia**

Exposure therapy is the most effective treatment for specific phobias, including biophobias. It has been shown to outperform wait-list controls, placebo treatments, and other active treatments (Wolitzky-Taylor et al. 2008). Exposure therapy involves repeatedly confronting one's fear in the absence of harm until the individual learns that their feared stimulus does not pose a risk. Fear can be confronted through imagination, virtual reality, or computer-assisted programs, though real-life exposure is generally the most effective in the short term (Wolitzky-Taylor et al. 2008, Bandelow et al. 2022). Exposure therapy is highly efficient, because a single extended session (2–3 hours) followed by regular, natural exposure in daily life can lead to drastic reductions in fear (Odgers et al. 2022).

Unfortunately, individuals with animal phobias are less likely to seek treatment than those with non-nature-related specific phobias (Wardenaar et al. 2017). One reason for low treatment-seeking behavior is that not everyone recognizes irrational fear as senseless, even when it is accompanied by significant avoidance or impairment in personal, social, or occupational/academic functioning that warrants a specific phobia diagnosis (Grenier et al. 2011). Only half of those who seek treatment for a specific phobia find it helpful (de Vries et al. 2021). This may be because, despite strong evidence for the superiority of exposure therapy, many therapists are not trained in delivering it, and when they have been, they may adapt it in an overly cautious manner that reduces its effectiveness (Trivasse et al. 2020). Ineffective treatment can prolong suffering and discourage further help-seeking, which is disheartening, because specific phobias are among the most treatable mental disorders (Norberg et al. 2024).

As a corollary, it is worth mentioning that there is little evidence supporting the use of medication for treating biophobias (Diemer et al. 2013). Although benzodiazepines may be prescribed to quickly reduce acute anxiety in one-off situations (e.g., a trip to the Amazon Rainforest), they have been shown to be ineffective over the long-term and may even increase anxiety over time (Wilhelm and Roth 1997, Essau et al. 2000, Thom et al. 2000). In addition, one-third of patients who are prescribed benzodiazepines may end up using them for more than a few weeks, which may lead to tolerance, dose escalations, and withdrawal reactions, such as increased anxiety, distractibility, sleep disturbances, and memory loss (Reid Finlayson et al. 2022).

Beyond their direct effects on mental health, biophobias reduce interactions with nature, limit outdoor activities, and drive the persecution of biophobia-inducing organisms, ultimately harming those organisms and the broader environment (Soga and Gaston 2016). For instance, the use of insecticides, repellents, traps, and sanitizers in households can negatively affect the health of all living beings in and around the home. Similarly, the use of chemicals in natural environments and agricultural settings results in fauna mortality, environmental pollution, and leaves chemical residues on crops intended for human consumption (Hart and Pimentel 2002, Poudel et al. 2020). Biophobias can also diminish public support for the conservation of certain species or habitats as people do not want to protect what scares or disgusts them (Knight 2008, Soga and Gaston 2024). These examples highlight the complex interconnections between human societies and ecosystems and the broad-ranging, diverse, and sometimes overlooked problems associated with biophobias.

In recent years, similar problems involving the mutual health of people, wildlife, and the environment—such as infectious diseases (Cunningham et al. 2017), antimicrobial resistance (Robinson et al. 2016), and food safety (Garcia et al. 2020)—have been successfully approached through the lens of One Health (WHO 2017, Zinsstag et al. 2021). One Health is a multidisciplinary paradigm that recognizes the interconnectedness of the health of humans, wildlife, and ecosystems, whereby issues affecting one domain can have significant impacts on others. Therefore, the One Health approach advocates collaborative efforts across various sectors and disciplines—including public health, veterinary medicine, ecology, conservation biology, and agriculture—to address complex planetary health and environmental challenges (WHO 2017).

We propose that biophobias are a quintessential One Health problem and should be studied through this lens. By relying on the International Classification of Diseases and the Diagnostic and Statistical Manual of Mental Disorders criteria for specific phobia to describe the pathology associated with biophobias, researchers have limited themselves to studying personal, familial, social, educational, and occupational impairments and have neglected the impact biophobias have on ecosystems and other living organisms. Furthermore, because many people find it easy to avoid their irrational fears by steering clear of nature or harming the natural world (e.g., using insecticide), biophobias have been given limited attention. In the present article, we provide examples on how the diverse impacts of biophobias can be mapped into a One Health scheme (figure 2). We also discuss knowledge gaps that need to be addressed to advance biophobia research and policy. Our goal is to guide the development of strategies aimed at mitigating the societal, economic, and environmental impacts of biophobia.

**Figure 2.**
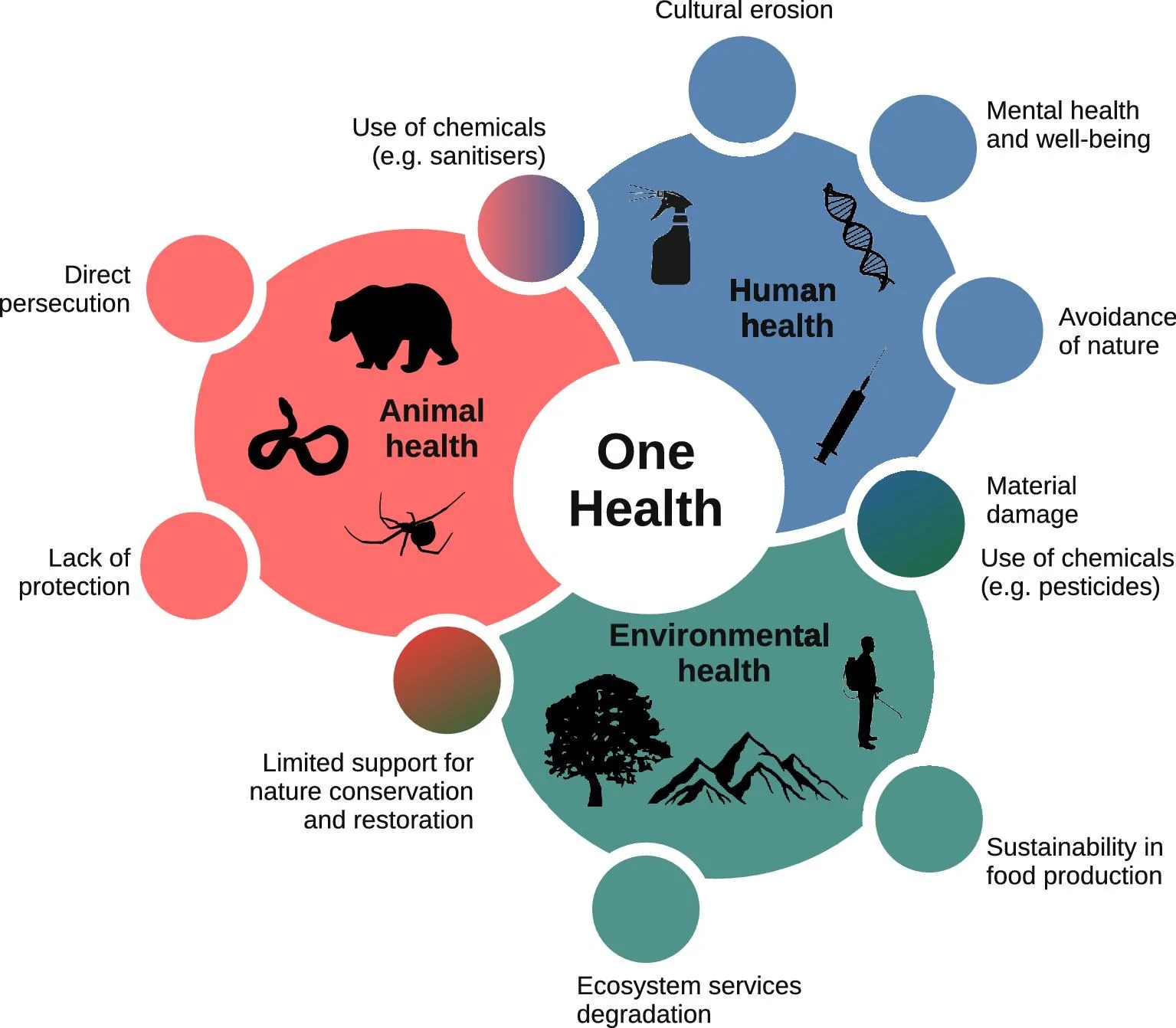
Reframing biophobias as a One Health problem. Different biophobias and their socioeconomic outcomes can be mapped onto one or multiple pillars of the One Health paradigm: wildlife, human, and environmental health (see some examples in figure 3). This approach enables the identification of specific areas for study and intervention that, even if initially focused on a single One Health pillar, may have ripple effects that influence the others.

## Wildlife health

Biophobias directly affect wildlife health through the persecution of species that trigger these phobic reactions. This leads to conflicts between humans and wildlife, especially when people engage in harmful behavior to control or eliminate organisms they find repulsive, such as applying chemicals (e.g., insecticides and pesticides) or outright killing animals such as insects, rodents, snakes, and even larger wildlife that enter human spaces. For example, many vulture species are facing extinction because of widespread persecution and unintentional poisoning (Buechley and Şekercioğlu 2016, Santangeli et al. 2022). This, in turn, is causing changes in scavenger communities, with an increase in opportunistic species such as feral dogs and rats, leading to negative health and economic impacts (Ogada et al. 2012). Therefore, wildlife persecution not only drives population declines for individual species but may also disrupt ecosystem dynamics, creating broader environmental health issues (see the “Environmental health” section).

Biophobias may play a role in shaping the human dynamics that drive support for species protection (Knight 2008, Small 2021, Soga and Gaston 2024). Conservation campaigns frequently rely on public support and donations, and when the species in need of protection are not appealing to the public, they are often sidelined. There is growing evidence of uneven conservation efforts toward species, with the most “charismatic” organisms often receiving the lion's share of research interest and conservation funding (Mammola et al. 2020a, 2023, Clark and May 2002, Adamo et al. 2022). This disparity in conservation priorities may inadvertently accelerate the decline of biophobia-inducing species (e.g., bats, Kingston 2016; spiders, Milano et al. 2021; insects, Simaika and Samways 2018; and snakes, Ceríaco 2012), undermining broader efforts to preserve biodiversity. Quantitative evidence for this, however, remains limited, and there is a general lack of studies yielding causal attribution (Soga and Gaston 2024). Importantly, these dynamics extend beyond biodiversity conservation efforts and apply also to restoration efforts. For example, biophobias can be a barrier to nature rewilding efforts (Bauer et al. 2009) or to the establishment of *wild* or *savage* gardens (i.e., gardens that aim to mimic the conditions and aesthetics of natural ecosystems) and other actions aimed at promoting biodiversity in urban environments (Russo 2024).

## Environmental health

Reactions to biophobias can accumulate, driving broader environmental impacts. Prototypical biophobic predators such as snakes, spiders, or large carnivores are critical to maintaining balance within ecosystems (Sergio et al. 2008). The decline or absence of these predators due to human persecution can disrupt food webs and result in unchecked growth of pest species, leading to agricultural damage, increased disease transmission, and further biodiversity loss. Similarly, the indiscriminate use of chemicals such as pesticides or disinfectants to eliminate biophobia-inducing organisms frequently harms nontarget species (Iyaniwura 1991, Aktar et al. 2009, Marteinson et al. 2022). Birds, mammals, and pollinators are often unintentionally affected, which can lead to further imbalances in food webs. Pollinators, for example, are crucial to the reproduction of many crops. Therefore, the decline of pollinator populations not only threatens biodiversity but also endangers global food production. Moreover, heightened biophobia can lead to the removal of natural environments, such as shrubs in gardens or parks, which negatively affects ecosystems as a whole.

These harmful activities may extend beyond visible species. Germ phobia (i.e., mysophobia) can lead to the overuse of sanitizers, detergents, and disinfectants, which alters natural microbial communities. Diverse microbial communities play a key role not only in human health but also in nutrient cycling, plant health, and overall animal health. Therefore, shifting the composition of these microbial communities can negatively affect both the health of hosts and the environment. For example, the steadily increasing use of sanitizers can lead to unintended consequences, such as changes in the composition and functioning of water microbiota (Wang et al. 2021).

Biophobias can also carry costs and consequences in terms of environmental sustainability. One clear example is the unwillingness to incorporate insects into Western diets, which is seen as a major barrier to the adoption of sustainable, alternative protein sources (La Barbera et al. 2018, Sogari et al. 2023). Insects offer a highly sustainable source of protein that requires far fewer resources—for example, land, water, and feed—than conventional livestock. In addition, insect farming generates lower greenhouse gas emissions and has a smaller ecological footprint overall (Van Huis and Oonincx 2017). Therefore, overcoming entomophobia could open new pathways for reducing the environmental pressures of food production, such as deforestation, habitat loss, and high carbon emissions linked to conventional livestock farming.

Another example is the potential barrier biophobias pose to efforts aimed at improving environmental sustainability through design. Indeed, biophobias are thought to be one of the main limitations to biophilic design efforts, which seek to leverage human affinity with natural systems and processes to improve the sustainability and design of buildings and other products (Wijesooriya and Brambilla 2021). Biophilic design involves features inspired by nature (i.e., biomimicry) or the inclusion of more biodiversity in the designed spaces (Heerwagen and Hase 2001). Biophobias can lead not only to the rejection of specific organisms or landscape features (such as the wild gardens example provided in the previous section; Russo 2024) but also the dismissal of the design elements they inspire. For example, many people reacted negatively to aspects of the clustered app menu design in the 2015 version of the Apple Watch and the cluster of camera lenses of the 2019 version of the iPhone. These negative reactions were associated with trypophobia, which is largely driven by strong feelings of disgust toward clusters of small holes typically seen in natural elements such as beehives and the compounded structure of some fruits (e.g., durian, strawberries). Trypophobia is thought to have an evolutionary basis related to aposematic patterns or disease avoidance—that is, a general aversion to stimuli associated with toxic or venomous animals or infectious diseases (Thiebaut et al. 2024). This example illustrates how consumers’ reactions may lead to negative environmental outcomes if they result in the rejection of sustainable design choices or increased product waste.

Last but not least, increased biophobia can reduce interactions with nature. This phenomenon, termed the *extinction of experience* (Soga and Gaston 2016), is known to decrease a wide range of proenvironmental behaviors, such as making environmental donations, purchasing ecofriendly products, and conserving water and energy (Soga and Gaston 2024). Therefore, through these indirect pathways, heightened biophobia can hinder efforts to address many of today's pressing environmental challenges.

## Human health

Biophobias affect the health and welfare of humans. Some individuals go to such great lengths to rid their environment of organisms that induce biophobia that they engage in dangerous practices, occasionally resulting in accidents such as setting their house on fire (e.g., attempting to exterminate spiders or insect colonies using blow torches or other hazardous tools; Gott 2020, Mammola et al. 2022). Although these extreme reactions and consequences are rare, people's emotional responses and avoidant behavior more commonly result in functional impairment. One in five individuals with a specific phobia experience severe role impairment, averaging 29 days out of role per year (Wardenaar et al. 2017). Severe impairment is most often experienced within the home, whereas social and relationship difficulties tend to be more pronounced but slightly less common (Wardenaar et al. 2017). Importantly, those around the sufferer are often also affected (Norberg et al. 2024). Parents and caregivers may accommodate a child's phobic behavior to reduce their anxiety, but this often places added strain on their own lives. Adults with phobias may also expect others to deal with feared objects and situations for them. For example, they might ask others in the household to kill a cockroach or take over activities that could involve encountering the phobic stimulus, such as doing yard work. Over time, relationships may become strained because of these demands and added responsibilities. Extensive avoidance and impairment may require treatment, which is not only costly but often also difficult to obtain (box 1).

Beyond the direct health impacts of medical and psychological treatments, biophobias can have significant indirect effects on human health. For example, the aversion to organisms such as insects, spiders, and microorganisms often leads to collateral health consequences through the increased use of insecticides and sanitizers. These health effects can be especially pronounced among individuals afraid of or disgusted by germs, because exposure to chemicals or the disruption of healthy microbiota may result in conditions such as skin rashes (Velazquez et al. 2019). There may also be broader societal implications, as is described by the hygiene hypothesis, which suggests that reduced exposure to microbes (e.g., through heightened disinfection) may alter the human immune system and contribute to the rising incidence of autoimmune and allergic diseases (Strachan 1989). The overuse of such chemicals has, in part, been experimentally linked to biophobias (Gish 2024, Gish et al. 2024). For instance, an online survey of 2500 individuals conducted by Gish and colleagues (2024) showed that both a heightened disgust response and limited knowledge about arthropods were significant predictors of insecticide use.

Biophobias also contribute to nonuse values, such as cultural erosion and avoidance of nature, both of which are connected to human health and may mutually affect each other. Increasing evidence suggests that the high incidence of biophobias may stem from the gradual disconnection between humans and nature (Soga and Gaston 2016, Robinson et al. 2021, Beery et al. 2023). This trend is particularly pronounced in highly urbanized areas, where opportunities to encounter nature are reduced (Hand et al. 2017, Fukano and Soga 2021, Correia and Mammola 2024). Over time, this pattern is likely to create a self-perpetuating cycle, with reinforcing feedback loops in which heightened biophobia leads to intensified negative sentiments toward nature and decreased outdoor activity (Soga et al. 2023). For example, less time in nature can reduce human contact with natural bacteria that are essential to the establishment of a healthy microbiome and immune system (Robinson and Jorgensen 2020). Avoidance of the outdoors may also erode cultural practices. For example, insect catching has traditionally been a long-standing hobby in Japan (Kawahara 2007); however, many children today no longer engage in this activity (Soga and Gaston 2016).

## The potential of One Health thinking in studying and tackling biophobias

In conservation science, the focus is frequently on the wildlife and environmental health pillars of the One Health framework, whereas psychiatry concentrates almost exclusively on the human health pillar. This disconnect often results in a partial understanding of the problem at hand. Adopting a One Health perspective towards biophobias and understanding and mapping how they affect the three pillars of wildlife, environmental, and human health may yield new insights and beneficial consequences. Foremost, this can serve as an effective communication and awareness-raising tool, promoting transversal thinking and emphasizing that what may seem like a sector-specific problem is rarely isolated (figure 3). It helps illustrate the important but often overlooked fact that addressing a given biophobia has broader implications for both human and planetary health.

**Figure 3.**
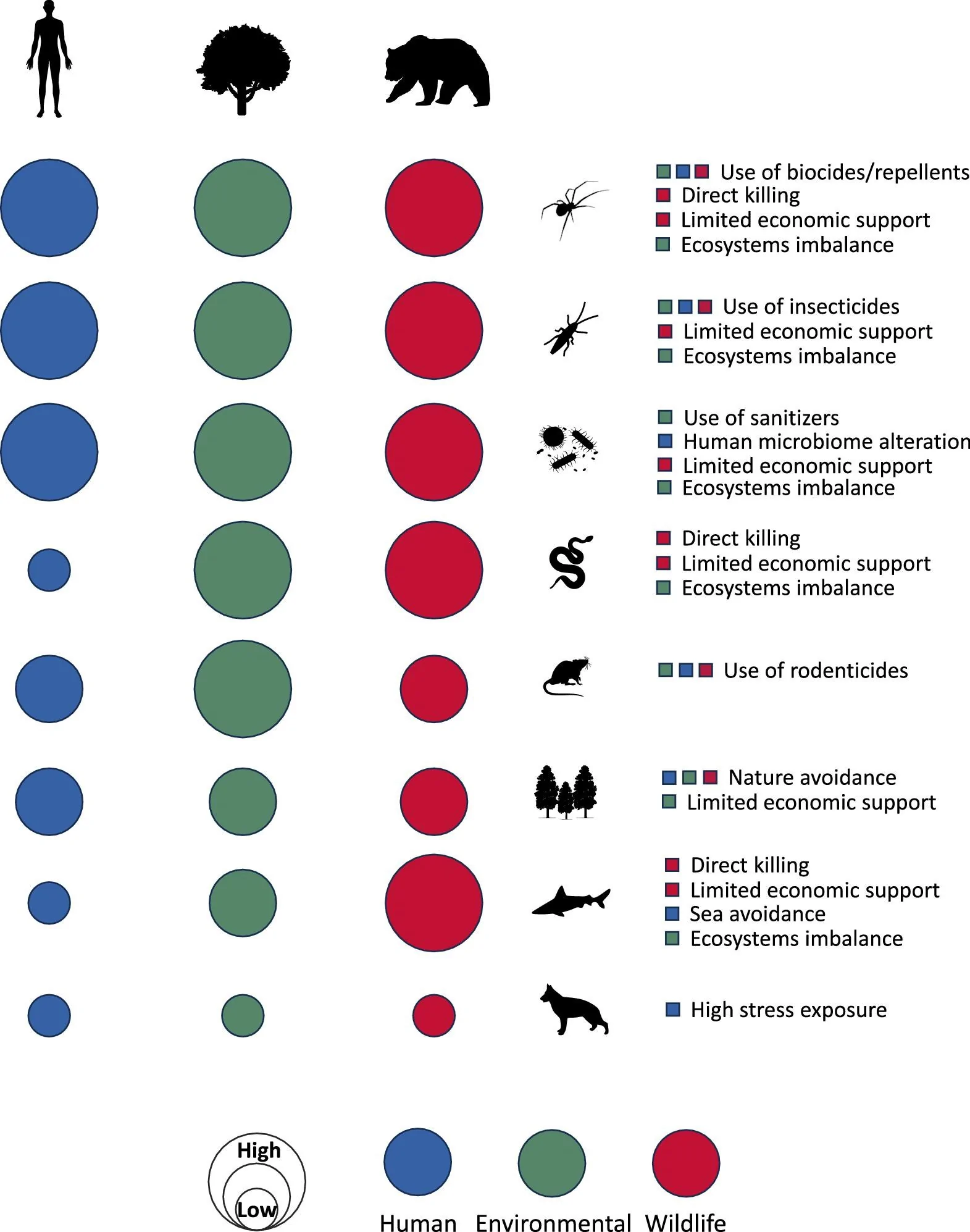
Method for displaying impacts of different biophobias in a One Health context. The figure illustrates a potential method for weighting the impact of different biophobias (listed in figure 1) through a One Health approach. The columns represent the three components: human, environmental, and wildlife health. Each row represents a specific biophobia. For illustration purposes, the size of the circles (low, medium, high) reflects the negative impacts of each biophobia across the three pillars of One Health. Note that all values in the figures are determined by our expert judgement for illustration purposes; research is required to rigorously test these assumptions to determine their validity and understand the full impact of biophobias. Images: *Homo sapiens* by Katy Lawler (CC BY 4.0); *Latrodectus hasselti* by Cathy (CC BY-SA 3.0); all other silhouettes under public domain (CC0).

On a more applied level, the interconnectedness of the three pillars, although it is challenging to study comprehensively, becomes a strength when designing targeted actions to address the issue. By studying biophobia as a One Health issue, we can better understand the reciprocal effects of human behaviors driven by anxiety and irrational fears on wildlife health (e.g., persecution), ecosystem health (e.g., damaged by unnecessary exterminations), and public health (e.g., affected by both mental health issues and environmental consequences). For instance, conservation scientists often lament the lack of protection for overlooked or uncharismatic organisms (Kunich 2000, Cardoso 2012, Mammola et al. 2020b, Wade 2020, Adamo et al. 2022), but these concerns rarely lead to significant changes in the status quo. A more effective approach might involve focusing on the human health pillar, specifically by reducing biophobic attitudes through education, exposure, and therapy (Soga et al. 2020). For example, reducing phobic sentiments toward insects can be expected to lead to a decrease in insecticide use and increase people's willingness to support insect conservation (Knight 2008, Simaika and Samways 2018, Samways et al. 2020). This, over time, would contribute to healthier food, promote biodiversity, and help preserve essential ecosystem activities provided by diverse invertebrates (e.g., Dangles and Casas 2019, Cardoso et al. 2025). Similarly, interventions aimed at reducing the prevalence of various biophobias may indirectly encourage more people to engage in nature-based activities.

## Moving forward a One Health agenda for biophobias

Biophobias, by influencing everything from biodiversity, to human health, to sustainability, impose an underappreciated burden on human societies and ecosystems, with far-reaching consequences. One Health thinking could guide research and policy efforts that integrate the multiple dimensions of biophobias and better communicate their broader impacts for society. However, the true extent of these impacts has yet to be systematically addressed and fully quantified, with most of the evidence linking biophobias to their impacts being correlational. To establish causality, a combination of controlled experimental and longitudinal studies is necessary. Our limited understanding constrains the development of policies and actions that can minimize the negative consequences of biophobias. To move a research agenda forward and make it more concrete, two main knowledge gaps must be addressed.

### How can we better quantify the socioeconomic dimension of biophobias?

We do not fully understand whether certain pillars of the One Health framework are more economically, environmentally, or culturally affected by biophobias than others. Although not all negative consequences of biophobias will be easily quantifiable and expressed in monetary terms, one option to better understand them would be to assess their economic costs. This would involve systematically gathering existing data on medical expenses, pest control costs, property damage, and marketing losses associated with biophobias. It would also include, if available, quantifiable nonmarket benefits—expressed in terms of health, recreational, and cultural value—lost because of the lack of use and interaction with nature (box 2). Such an effort would provide a comprehensive view of the global economic burden of biophobias and could help guide policy development and resource allocation. Furthermore, it would allow tracking whether socioeconomic costs associated with biophobia are varying across regions, time periods, and cultures. For example, in regions or cultures where pest species are perceived as a greater threat, the cost of controlling these species may be higher. Conversely, in more urbanized areas, costs may be more related to mental health or property damage. Importantly, longitudinal studies could track how these costs evolve in response to changing environmental conditions, such as climate change, which may alter the distribution of species that trigger biophobia.

Box 2. **Measuring the economic impacts of biophobias**

Studies on the costs of biophobias are limited. European and US research on anxiety disorders, including specific phobias, suggests that about 67% of costs stem from productivity losses, with 33% due to direct healthcare expenses (Andlin-Sobocki et al. 2005, Olesen et al. 2012). Indirect costs, such as sick leave, generally surpass direct healthcare costs. For example, animal-related phobias incur costs of roughly €800 per patient per year, with direct healthcare making up 44% of this amount. All these studies assessed damages and losses in economic terms by considering direct healthcare costs, non-medical costs (e.g., nursing), and indirect costs (e.g., absenteeism and damage to property), a model that could be applied to biophobias.

Additional economic impacts of biophobias are related to the downregulation of ecosystem services, which are often quantified in economic terms but not included in market transactions (Brander et al. 2024). Examples include the valuation of changes in crop production due to a reduction in pollinators’ abundance or diversity (Porto et al. 2020) and the economic quantification of pest control services provided by bats in acroecosystems (Boyles et al. 2011, Frank 2024).

Instead, reductions in benefits related to “non-material” values, such as fear, anxiety, and even disgust toward animals (Adler 2004)—all of which affect the quality of life and, indirectly, the environment—cannot be readily addressed through market-based approaches. These values can, however, be assessed using stated preference methods, such as the contingent valuation method (Bateman and Turner 1992, Hanemann 1994). This method involves surveying respondents to measure fear on a well-being scale, such as the quality-adjusted life year, and asking them to state their willingness to pay to avoid reductions in well-being (Adler 2004). Nonuse values—for example, those associated with the appreciation of wildlife protection for the benefit of others (altruistic value) or for its intrinsic worth (existence value)—may be quantified by eliciting people's preferences through direct interviews or choice experiments. Although we lack studies on biophobias, measures based on willingness to accept compensation could potentially value coexistence with phobia-inducing animals, representing economic costs of conservation.

New approaches relaxing assumptions of mainstream economics (Lawson 2013) that people have pre-formed preferences in eliciting values, may be used to generate values on the basis of deliberative monetary valuation (Kenter et al. 2016). Under this approach, preferences on the conservation of nature can be elicited using deliberative strategies where the final measure of the willingness to pay or accept for nature protection is endogenously generated through discussion rather than being assumed as pre-formed. Limited examples exist for the valuation of biodiversity using this approach (Martino and Kenter 2023), but we envision that it may be adopted to measure the effect of biophobias connected to One Health. The endogenous construction of values proposed by the deliberative monetary valuation may be used to support the treatment of biophobia by facilitating through a dialogic way the analysis of fears of and preferences on wilderness between patients and between patients and medical specialists.

### What policies and actions are most effective in reducing the impact of biophobias?

The few existing policies aimed at mitigating the effects of biophobia are often reactive (e.g., focused on pest control) rather than proactive (e.g., trying to address the root causes of fear). What is needed is a set of preventive, educational, and regulatory policies and interventions that reduce both the prevalence of biophobia and the associated costs. For example, whereas regulations limiting pesticide use could help mitigate environmental damage in the short run, increasing public awareness campaigns that highlight the ecological benefits of insects could reduce persecution costs and encourage more sustainable practices in the long term (Samways et al., 2020). More generally, educational programs—especially those integrated into school curricula—could be one of the most effective long-term strategies for reducing biophobias. All of these policies, however, need to be rigorously scrutinized over time to assess their effectiveness.

## Conclusions

Although the importance of biophilia (Wilson 1993) in fostering sustainable human–nature relationships is widely recognized, the impact of biophobia has received surprisingly little attention outside of psychiatry. However, as is outlined in the present article, biophobia can lead to a range of negative consequences affecting multiple dimensions of human societies and ecosystems. It is predicted that biophobias may escalate in the foreseeable future because of growing global urbanization and the resulting loss of direct experiences with nature. Despite this, progress in the study of biophobias has been uneven and unstandardized, lacking a coherent framework for development. This absence of theoretical grounding may limit the focus on policies and actions aimed at mitigating the growing prevalence of biophobia. With this in mind, we illustrated the potential of using a One Health approach to better understand the multidimensional impacts of biophobia across societies and ecosystems. Expanding research in this area, and addressing the key challenges highlighted, will be crucial for developing a sustainable relationship between humans and the natural world.

## Author Biography

Stefano Mammola (stefano.mammola@cnr.it, stefano.mammola@helsinki.fi) is affiliated with the Molecular Ecology Group at the Water Research Institute at the National Research Council in Verbania, Italy; with the Finnish Museum of Natural History, at the University of Helsinki, in Helsinki, Finland; and with the National Biodiversity Future Center, in Palermo, Italy. Veronica Nanni is affiliated with the Molecular Ecology Group, at the Water Research Institute, at the National Research Council, in Verbania, Italy, and with the Science, Technology, and Society Department at the School for Advanced Studies, in Pavia, Italy. Simone Martino is affiliated with the James Hutton Institute, in Aberdeen, Scotland. Ricardo Correia is affiliated with the Biodiversity Unit of the University of Turku, in Turku, Finland; with the Helsinki Lab of Interdisciplinary Conservation Science, Department of Geosciences and Geography, at the University of Helsinki, in Helsinki, Finland; and with the Helsinki Institute for Sustainability Science, at the University of Helsinki, in Helsinki, Finland. Ester M. Eckert is affiliated with the Molecular Ecology Group, at the Water Research Institute, at the National Research Council, in Verbania, Italy, and with the National Biodiversity Future Center, in Palermo, Italy. Melissa M. Norberg is affiliated with the Lifespan Health and Wellbeing Research Centre, at Macquarie University, in Macquarie Park, New South Wales, Australia. Masashi Soga is affiliated with the Graduate School of Agricultural and Life Sciences, at the University of Tokyo, in Tokyo, Japan.
